# Exploring Sensory Attributes in Spinach- and Offals-Filled Chicken Roulades: An Empirical Analysis

**DOI:** 10.3390/foods14020303

**Published:** 2025-01-17

**Authors:** Paul-Corneliu Boișteanu, Bianca-Georgiana Anchidin, Marius-Mihai Ciobanu

**Affiliations:** 1 Faculty of Food and Animal Sciences, “Ion Ionescu de la Brad” Iasi University of Life Sciences, 8 Mihail Sadoveanu Alley, 700489 Iasi, Romania; paul.boisteanu@iuls.ro; 2 Department of Food Technology, ‘Ion Ionescu de la Brad’ Iasi University of Life Sciences, 700490 Iasi, Romania

**Keywords:** chicken roulades, sensory analysis, check-all-that-apply, principal component analysis, preference mapping, penalty analysis, spinach, offals

## Abstract

One of the most significant industries in the world is the meat sector, and development into new goods is ongoing due to high customer demand and fierce market competition. Products made from chicken are widely favored. This phenomenon can be attributed to the lack of cultural or religious restrictions on this meat. The study looks into how using two distinct types of iron-rich fillings impacts the sensory profile of classic chicken roulades. The purpose of the study is to determine how changes in sensory qualities (such as taste, texture, and flavor) affect product acceptance among customers. This approach uses methods like check-all-that-apply (CATA), principal component analysis (PCA), external preference mapping (PrefMap), and penalty analysis to explore the impact of adding chicken offals and spinach as fillings on the sensory profile of traditional chicken meat roulades. This approach seeks to expand the meat product category, create new goods that use both plant and animal components, invent new ways to use offals and spinach in the market, and ensure that consumers accept these new products. Based on the results of our investigation, the type of filling utilized in the roulades showed the most differences across all sensory tests. According to hedonic analysis and preference mapping (PrefMap), the majority of customers favored roulades with spinach fillings as opposed to those containing chicken offals, which only a small portion of customers liked. Variations in consumer preference for roulades filled with chicken offals were noted in the anatomical region, with slightly greater preference for roulades made from chicken breast. However, this (anatomical region used) did not significantly affect the outcomes of all sensory tests. The spinach stuffing was found to be quite popular with the customers, even outperforming traditional chicken roulades, making it the most significant influence. Based on consumer appreciation, this suggests that the spinach-filled chicken roulades may be a viable option for meat products in the future.

## 1. Introduction

Food products have been accepted or rejected based on sensory assessment since ancient times. However, as industry and processed foods grew in the previous century, it started to become an accurate science. Since the food business started preparing food rations for soldiers during World War II, and it was imperative that they taste well, it saw a sharp rise. As a result, several sensory approaches were developed, and our understanding of human perception advanced [1,2]. Descriptive analysis (DA) using highly skilled assessors has been the norm for sensory characterization for many years [3,4].

To obtain a thorough qualitative and quantitative description of a product’s sensory qualities, sensory characterization is utilized. According to [5], it is among the most effective and frequently utilized instruments in sensory research. The subjective nature of sensory analysis stems from the human element [6]. It makes use of scientific concepts from physiology, statistics, psychology, and food science. Its goal is to use the panelists’ senses—sight, smell, taste, touch, and hearing—to evoke re-referenced reactions to sensory aspects of food, such as texture, flavor, taste, appearance, and smell [7,8]. Data gathered from human perception often reveals a considerable degree of participant diversity (cultural, educational, environmental, habits, limitations, variety in sensory skills and preferences, etc.). This sort of study is unable to control for many individual liabilities [5,6]. Consumers place a premium on foods’ sensory qualities (taste, texture, smell, etc.), and the innovation process needs to take these into consideration because the consumer’s decision to buy a product or stick with it depends on these qualities. Market success and product quality may be greatly enhanced by further research on stakeholder needs in final product applications, such as sensory analysis and consumer certification [6]. Due mostly to population growth and affluence, it is anticipated that the world’s consumption of animal protein would rise by 14% by 2030 when compared to the average for the 2018–2020 base period. By 2030, 17.8% of chicken protein will be available. Consumption of meat has changed to include chicken. This shows that chicken meat is less expensive than other meats in low-income developing nations, while white meat is more popular in high-income nations due to its ease of preparation and perceived health benefits [9]. Chicken meat accounts for more than 70% of the world’s poultry consumption, making it the second most popular meat in the world today [10]. According to projections, chicken will make up 41% of all animal protein by 2030, which is a two percentage point increase over the base year (2018–2020). The percentage of meat from other animals is smaller worldwide; according to [9], sheep make up 5%, beef 20%, and pork 34%. This suggests that a sizable volume of chicken slaughter by-products is created daily at slaughterhouses.

Internal organs, such as the heart, liver, spleen, and kidneys, which make up a sizable amount of a chicken’s live weight, are typically among the edible products obtained from chicken slaughter. Yields vary from 5 to 6%, depending on the age of the animal [11,12]. Meat processors can use the plentiful supply of edible products made from chicken carcasses to boost economic profitability while lowering losses, even though these products make up a sizable portion of the live weight and are crucial ingredients in culinary preparations [12]. Because there is no lucrative market, by-products from carcass cutting (organs) are seen unfavorably. Since the yield of by-products for chickens is between 5 and 6% of live weight, increased attention should be paid to by-products, especially since most of these products offer a diverse range of foods that are nutritionally appealing, having a high protein content and good nutritional properties due to the presence of many essential nutrients and having a wide variety of flavors and textures [13,14]. Chicken liver and gizzards are richer sources of protein (17.70% and 17.26%, respectively), comparable to the protein levels of pork but slightly lower than those of chicken and beef [15]. Furthermore, compared to other edible chicken parts, the liver has greater concentrations of all vitamins. Additionally, it has been noted that the largest concentration of vitamin A is found in the heart of chickens, as opposed to the organs of sheep, beef, and pigs [16,17]. Furthermore, compared to meat-derived products and muscle tissues discussed in the specialist literature, the majority of chicken organs contain greater amounts of total unsaturated fat and polyunsaturated fat and lower levels of total saturated fat. Even more impressively, all of the resulting compounds had favorable EAA/AA and PUFA/SFA ratios [12]. Today, the organization of the production of high-quality meat products is one of the most urgent tasks in the meat industry. The use of various types of plant ingredients in the development of these preparations can significantly reduce their calorie content and recommend them as “healthy” food products, which are becoming increasingly popular among consumers [18].

High levels of minerals, including iron (Fe), calcium (Ca), and zinc (Zn), may be found in leafy greens. Moreover, they are rich in vitamins E, K, B, and C, as well as beta-carotene [19,20]. Additionally, if leafy greens are grown and processed properly before consumption, some of the anti-nutritional components (like oxalate, phytate, and glucosinolates) will be reduced [19,20,21,22,23].

This study focuses on consumer perception and sensory evaluation of iron-enriched chicken roulades from both plant-based and animal-based sources to enhance the iron content of a naturally low-iron meat product. The aims of this study are represented by:

i. Realization of chicken roulades containing iron-rich fillings from two different sources: plant-based (spinach) and animal-based (chicken offal—chicken hearts, gizzards, and liver), which could be a novel meat product;
ii. Making chicken rolls with the same fillings from chicken meat from two different regions of the carcass: chicken breast and chicken thigh; in order to analyze their possible influence on the sensory profile of the products and the consumers’ perception;
iii. Sensory analysis of the products using descriptive and affective sensory tests.

## 2. Materials and Methods

### 2.1. Materials

Six types of chicken roulade were made for this investigation. Four of them were innovative items that had been enhanced with vegetal and animal ingredients, rich in iron, while two of these were used as a control sample. Chicken breast served as the foundation for half of the batches, while boneless chicken thigh served as the base for the other half. These two distinct anatomical sections were employed to assess their respective sensory peculiarities in relation to the final products’ sensory qualities and consumer perception. To the newly developed roulade variants, two kinds of ingredients were added: animal and vegetal. The animal ingredients consisted of chicken liver, hearts, and gizzards (SC Fermador SRL, Iași, Romania), and the vegetal ingredient was spinach (Edenia, SC Macromex SRL, Bucharest, Romania). Chicken offals, which stood in for the animal filling in the roulades, were also bought from the same local producer as the chicken meat, which was represented by chicken breast and chicken thighs (SC Fermador SRL, Iași, Romania). We bought the ground black pepper, pink and green peppercorns, garlic powder, sweet paprika, salt, and elastic food netting from Rocas FDS S.R.L. (Brătuleni, Iași, Romania). In Iași, Romania, a small shop sold the food film (Fino, Sarantis România S.A., Bucharest, Romania). Table 1 displays the percentage figures for each roll assortment’s composition.

### 2.2. Preparation of the Chicken Roulades

The chicken rolls were made in the didactic workshops of meat processing (initial meat processing and salting) and meat product preparation (the actual realization of the products) within the “Ion Ionescu de la Brad” Iasi University of Life Sciences (IULS) Iasi.

The boneless chicken breasts and thighs were trimmed to remove any flax (tendons, cartilage, etc.), cut to obtain the flattest possible pieces of meat, and dry salted by mixing with 1.36% salt. For good absorption of the salt deep into the meat, it was stored in a refrigerated maturing room for 24 h at 2–4 °C.

The filling for the roulades was made before the roulades were made and consisted of seasoned spinach (previously thawed at 2–4 °C) and chicken offals (liver, hearts, and gizzards; these were cleaned of any blood residues) with 0.56% salt and 0.11% ground black pepper. Chicken breast pieces (chopped with a cutter) resulting from the cutting of the meat were also added to the filling. One type of filling (approximately 28% of the total finished product) was added to the pieces of meat placed on a piece of pre-prepared food foil and rolled. The roulades were divided into 4 batches according to the anatomical region and the filling used and frozen (−18 °C) for 24 h to maintain their shape during the following technological operations: tying and stuffing. The 2 batches of control roulades were made in the same way; only they did not include fillings. Once the freezing operation was completed, the roulades were removed from the food film and tied with a food-elastic net to obtain a specific roulade shape during the heat treatment. The chicken roulades that were used as control samples were not filled with any filling at all, as there is no standard type of filled chicken roulade on the market, and we considered that the introduction of any type of filling in the control roulades would affect the sensory evaluation, sensory differences not being as easily detectable, as any type of filling would have affected the sensory profile of the product.

The tied roulades were passed through a bath composed of water, garlic powder, paprika, sweet paprika, and pink and green pepper powder (ground with a spice grinder) (Table 1) and hung on a stainless steel rack that was inserted into the heat treatment cell, the parameters of which are presented in Table 2.

### 2.3. Consumer Sensory Analysis

Students and teaching staff from the “Ion Ionescu de la Brad” Iasi University of Life Sciences comprised the panel of 32 semi-trained panelists, ages 20 to 42, who examined the six chicken roll samples in compliance with the guidelines of ISO 8586:2023 [24]. This age group was selected because it represents a sizable market segment for novel products, has the fewest food biases, and is the most receptive to innovation. Singh-Ackbarali et al. [25] and Marina et al. [26] are two studies that use university students for sensory testing in order to investigate youth acceptability, their behavior toward innovative cuisine, and their sensory development skills. The selection and training of sensory assessors for sensory analysis were conducted based on their prior experience, willingness to attend the two-month training sessions that preceded the final sensory analysis, and lack of health issues. This was given special consideration because it may affect the results of the sensory analysis. The two-month training was performed to guarantee that the tasters understood the products under analysis, the sensory tests that were employed, and the terminology. As demonstrated by Xiangli et al. [27], who conducted sensory analysis on a baijiu distilled beverage to examine the variations in the identification and scoring of its sensory qualities, this number of tasters is adequate to produce definitive results of sensory analysis. To examine the variations and connections between their sensory perceptions of baijiu samples, they employed a panel of 12 experts who are involved in the manufacture of baijiu and a panel of 21 semi-trained panelists for sensory analysis. The findings demonstrated that these taster groups’ sensory vocabulary (qualitative data) varied, with the semi-trained tasters describing commonplace phrases that they were familiar with but that had meanings that were strikingly similar to technical terms, and the expert tasters knowing more thematic and specialized terms. There were significant differences in the evaluation of professional sensory quality in the baijiu beverage production industry, but the quantitative data collected from both taster groups revealed that the majority of the sensory characteristics examined showed positive or negative correlations between the groups. This suggests that, despite the use of different lexicons, the two groups had strong commonalities in the sensory perception of baijiu samples. These findings highlight the necessity of standardizing sensory vocabulary, which is what we accomplished for the taster group in our study. As a result, tasters results are more consistent and linked. Additionally, the tasters in our case benefited from an explanation of the sensory terminology during the two months of taster training, which guarantees a link between the sensory outcomes. Another example comes from Giacalone and Hedelund [28], who used 11 semi-trained panelists to perform sensory analysis on eight chocolate samples. According to their findings, semi-trained tasters exhibit somewhat greater data variability than expert tasters; yet the panel-level consistency in evaluating sensory attributes lends credence to the application of this kind of panel for sensory research. The study’s findings confirm that the panel of tasters selection and training are crucial for the products under consideration. It appears that the training of the tasters is more significant than the panel size because both studies employ smaller taster groups than the one we utilized. To take part in the sensory analysis, each of the chosen panelists had to fill out a consent form that included details about the study’s goals, the methods that would be employed, and all of their rights as participants. All participants received and signed this document prior to the sensory analysis training, in accordance with ethical guidelines. Because standardization is essential for sample preparation and dissemination, the guidelines in ISO 13299:2016 [29] were adhered to. The six chicken roulade samples were presented in a randomized order, and each sample was given a unique three-digit code. In order to assess the consistency and reproducibility of the qualitative and quantitative data gathered, the tasting was conducted in two sessions over the course of two days. In order to minimize the potential impact of outside factors on the sensory analysis outcomes, both sessions were conducted under identical experimental settings. All of the chicken roll samples examined on the two days of the sensory analysis came from the same manufacturing batch in order to prevent any potential production modifications of a new batch of samples and to compare the findings and their statistical validity. The tasters were initially given samples as whole bars so they could fill out the questionnaire’s specific features. The samples were then cut into slices, about 1 cm thick, and given to each taster. In order to avoid changing sensory perception and preventing the mixing of sensory characteristics between samples, the panelists sampled products from all six batches of chicken roulade while adhering to the mouth rinse routine and the pause regime between samples. The XLSTAT program, version 2024.3 [30], was used for all statistical analyses in this investigation.

#### 2.3.1. Hedonic Ranking of the Products

In order to produce new food products and introduce them to the market, it is necessary to gauge whether or not the right customers find them appealing. Many rating scales have been created to gauge how much someone likes something [31,32]. Customers were asked to score their overall liking using a nine-point hedonic scale that was horizontal and anchored at “dislike very much” (1) and “like very much” (9) [33]. 

#### 2.3.2. Check-All-That-Apply (CATA)

In sensory and consumer science, the widely used check-all-that-apply (CATA) strategy has been gaining popularity. CATA offers a versatile multiple-choice question structure that is being used more and more for a variety of applications while not being a novel method. Participants in this study were given a predetermined set of descriptive features along with food samples to evaluate (Table 3). They had to choose every word they thought would be appropriate to describe the goods. As shown in the Table 3, the phrases used and their significance were then evaluated according to how frequently they were chosen by participants. The CATA method is comparatively simple to use, requires few instructions, and can be finished rapidly [34]. To demonstrate the connections between the sensory qualities and the assessors’ propensity to group these traits, the principal coordinate analysis (PCoA) test was conducted in addition to the CATA analysis.

#### 2.3.3. Multiple Correspondence Analysis (MCA)

The MCA’s primary characteristic, which serves as justification for its existence, is exemplified by the optimal scaling it attains: the coordinates of the attributes on the MCA graph’s axes are calculated to generate the greatest number of correlations (many multiples producing a complex result) between them, resulting in an extremely intricate graphical depiction of the relationships between variables [35]. Finding the connections between the various possible answers to the questions is the aim of an MCA. The information gathered from the panelists during the CATA study served as the foundation for the MCA analysis.

#### 2.3.4. Quantitative Descriptive Analysis (QDA)

The sensory qualities gathered by the panel of semi-trained tasters during the CATA questionnaire were scored in order to undertake the descriptive quantitative analysis of the data. The same panel of tasters then awarded these scores on a number scale from 1 to 9. After calculating the averages and standard deviations of the attributes for each of the six examined samples, the analysis of variance (ANOVA) was used to find differences between the examined samples.

#### 2.3.5. Principal Component Analysis (PCA)

The foundation of quantitative descriptive analysis (QDA) is the capacity to teach panelists how to measure particular product features in a repeatable way, resulting in a thorough quantitative product description that can be subjected to statistical analysis [36]. Using PCA, a dimension-reduction technique, a large number of variables can be reduced to a small set while retaining the majority of the information. PCA results are often interpreted in terms of loadings (the original variables), scores (original samples), and components, also known as factors (the transformed variable values corresponding to a data point) [37].

#### 2.3.6. Preference Mapping (PrefMap)

Sensory experts employ a variety of statistical approaches to model, examine, and comprehend the connections between product attributes and customer preferences. One such technique used to illustrate these connections is preference mapping. Based on intricate statistical procedures, this method creates two-dimensional maps of customer-like ratings [38]. The external preference mapping analysis was performed using the QDA analysis values for the 40 sensory attributes and the hedonic test scores. Based on their correlation, the panelists were grouped into clusters based on their individual preferences, and the products were positioned on the PrefMap graph, creating a graphical representation of the products and the consumers’ overall preferences.

#### 2.3.7. Penalty Analysis

A statistical technique that manufacturers most frequently employ to identify sensory aspects of goods that might not require modification is penalty analysis [39]. We chose to employ this method to determine which sensory characteristics of the six examined samples influence their ultimate hedonic score. The fundamental steps of this analysis were as follows: The panel of panelists evaluated the sensory attributes gathered in the CATA analysis and assigned each one a score on a scale of 1 to 5, where a score of 3 indicates that the characteristic is just about right (JAR), a score of 1 or 2 indicates that it is “not enough”, and a score of 4 or 5 indicates that it is “too much”.

## 3. Results

### 3.1. Consumer Sensory Analysis

#### 3.1.1. Hedonic Ranking of the Chicken Roulades Samples

Hedonic analysis, the primary indicator of whether a product is liked or disliked, is one of the most crucial processes in sensory analysis since, even when a product has positive sensory qualities, these may not all work well together. As a result, we made the decision to first gauge how much a product was enjoyed by the tasters after they consumed it, and we gathered information from the 32 semi-trained tasters for this evaluation. In order to examine the primary sensory elements of hedonic pleasure—appearance, smell, taste, and texture—in the rest of this article, we assessed the total score.

**Figure 1:**
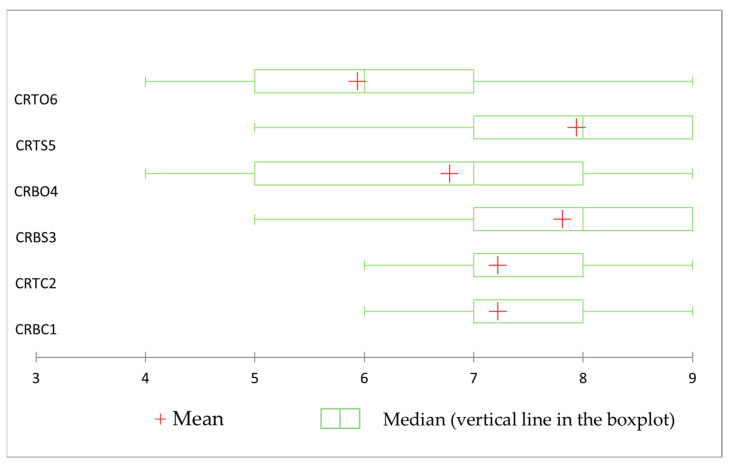
Graphical distribution of liking scores for chicken roulade samples. The scores were assigned on a scale from 1 to 9, where 1 represents the lowest level of liking and 9 represents the highest level of liking CRBC1—control chicken roulade made from chicken breast, CRTC2—control chicken roulade made from chicken thigh, CRBS3—chicken roulade made from chicken breast and spinach filling, CRBO4—chicken roulade made from chicken breast and chicken offals filling, CRTS5—chicken roulade made from chicken thigh and spinach filling, CRTO6—chicken roulade made from chicken thigh and chicken offals filling. The boxplot (the green rectangle arranged horizontally) represents the distribution of most of the hedonic test scores on the hedonic scale from 1 to 9, and the whiskers represent the minimum and maximum values obtained in the analysis.

The product scores are presented in Figure 1 in graphical form. In this figure, we can see the range of scores given to the products evaluated by the tasters. The lowest score obtained by a product (score of 4 on the hedonic scale) was one for the chicken roulade with offals (CRTO6), while the highest scores were obtained by the chicken roulades with spinach samples (CRBS3 and CRTS5). Also, the presence of large and equal-sized boxes indicates that the CRBS3 and CRTS5 samples scored extremely similarly in the hedonic tests, with the CRTS5 product exceeding the average score of the CRBS3 product by a very small amount, as indicated by the cross-shaped sign. The lowest score obtained for these samples of plant-based-added rolled with plant-based-added is a score of 5; however, these types of scores were exceptions given that the majority of the hedonic scores of these products were in the 7–9 range, as can be seen in Figure 1. As in the case of the spinach-filled rolls batches, the control chicken rolls batches (CRBC1 and CRTC2) also show very similar scores, with the majority of them in the 7–8 range and the minimum score obtained from the assessors being 6, which is higher than the minimum score obtained by the spinach-filled rolls (CRBS3 and CRTS5). According to the position of the whiskers at the ends of the boxes, we can notice that the evaluators had very similar answers, with no very obvious differences of opinion in their preference for these products. Regarding the chicken rolls filled with comestible organ meats (CRBO4 and CRTO6), consumers’ opinions are slightly more diverse, with the CRBO4 product being more preferred, as can be seen from the position of the box on the hedonic scale and its size. These two samples (CRBO4 and CRTO6) obtained the lowest scores from tasters during the hedonic evaluation. The tasters’ evaluations were also slightly more diverse here. These samples were least preferred by consumers, with scores falling mostly in the range of 5–7 (CRTO6) and 5–8 (CRBO4), which are the lowest of all the samples analyzed.

#### 3.1.2. Check-All-That-Apply (CATA)

**Figure 2:**
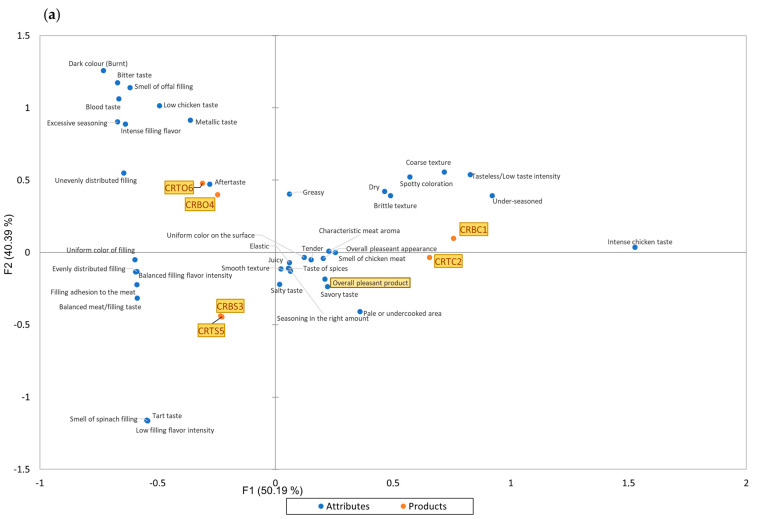

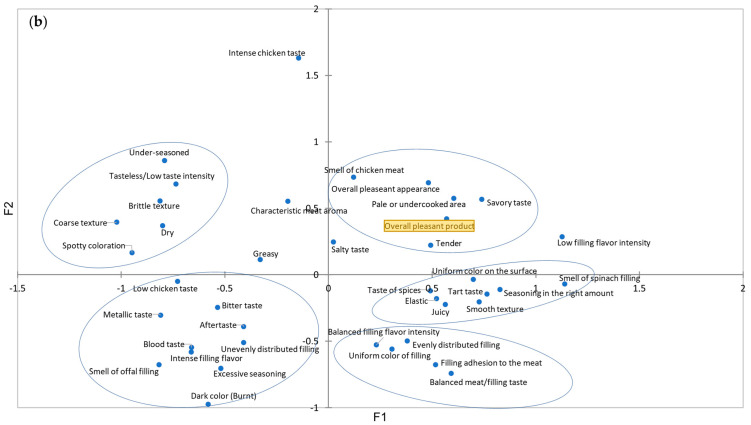
Graphical representation (symmetric biplots) of the check-all-that-apply (CATA) analysis for the six chicken roulades samples. (**a**) Check-all-that-apply (CATA) analysis displaying a bidimensional plot of the data on the F1 and F2 axes, which explain 50.19% and 40.39% of the total variance, respectively. CRBC1—control chicken roulade made from chicken breast; CRTC2—control chicken roulade made from chicken thigh; CRBS3—chicken roulade made from chicken breast and spinach filling; CRBO4—chicken roulade made from chicken breast and chicken offals filling; CRTS5—chicken roulade made from chicken thigh and spinach filling; CRTO6—chicken roulade made from chicken thigh and chicken offals filling. Sensory attributes located near samples suggest that they are perceived to be present for those samples. (**b**) Principal coordinate analysis of the relationships between CATA attributes. Characteristics that are adjacent to one another and, more specifically, in the same circle indicate that the tasters checked them together the most frequently. Overall pleasant product is the overall score obtained in the hedonic analysis, and its ranking illustrates which sensory most contributed to the greatest hedonic score.

The CATA questionnaire provided to the tasters was composed of 40 terms specific to the chicken roulades analyzed, chosen to reflect the specificity of sensory perceptions of both the filling and the general characteristics of the chicken roulades, thus providing a comprehensive, relevant, and complete analysis of the sensory profile of the products analyzed. Among the terms of the CATA questionnaire, 10 of them referred to sensory characteristics that were specific to the filling of the products, which were not specific to the control chicken roulades, or certain sensory characteristics that were specific only to roulades with spinach filling or only to roulades with organ filling (e.g., the smell of offals filling for roulades containing offals, the smell of spinach filling for products containing spinach). Because of this, sensory attributes that were considered irrelevant for a particular lot were not assessed within that lot. All the sensory attributes analyzed are present in Table 4, as well as the number of times they were present in all the samples analyzed. For greater accuracy of the CATA test results, the Cochran’s Q test was used to test for significant differences in the responses of this sensory test [40]. In addition to the Cochran’s Q test, the principal coordinate analysis (PCoA, Figure 2b) test in graphical form was also used to analyze the similarities between the sensory attributes of the CATA test. According to the results of the Cochran’s Q test presented in Table 4, the sensory attributes related to the taste of the samples (e.g., low filling flavor intensity, balanced filling flavor intensity, intense filling flavor, tasteless/low taste intensity, low chicken taste, balanced meat/filling taste, blood taste, bitter taste, savory taste, aftertaste), as well as those related to the texture of the samples (e.g., dry, juicy, smooth texture, elastic, filling adhesion to the meat), vary extremely significantly (*p* < 0.001) between them. Most often, there were highly significant variations (*p* < 0.01) in the samples’ olfactory perception (e.g., the smell of chicken meat, the smell of offals filling, the smell of spinach filling). The samples’ appearance displayed the most variability of any sensory property. While the attribute was present, it varied significantly (e.g., uniform surface color, patchy coloration), highly significantly (e.g., overall pleasing appearance, dark (burnt) color), extremely significantly (e.g., uniform color of filling, evenly distributed filling, unevenly distributed filling), and insignificantly (e.g., pale or undercooked area).

The graphs shown in Figure 2a,b are the results of applying the CATA statistical test. These graphs illustrate the relationships between the sensory attributes by graphically displaying the attributes that were chosen simultaneously within the same product. This is explained by the proximity of the points on the graph that represent the sensory attributes.

Together, the graph’s primary axes in Figure 2a account for 90.58% of the variance in the taster data. Based on the sensory qualities shown in CATA, the F1 axis displays the primary proportion of variance across the examined chicken roulade samples, accounting for the greatest portion of the overall variability. Complementary to axis F1, axis F2 displays the percentage of secondary variation in the examined samples. As can be seen from this figure, two of the samples are rather comparable in terms of their sensory characteristics. As with the CRBO4 and CRTO6 samples, CRBS3 and CRTS5 samples, and the non-filled samples (which serve as the control samples), CRBC1 and CRTC2 likewise exhibit similar clustering, which is created by products with the same sort of filling. The sensory characteristics that are positioned as near to the middle of the graph (where the F1 and F2 axes cross) as possible and extremely near to the overall pleasant product attribute—which is the hedonic test score that the tasters assigned to the examined samples—are regarded as positive. Among the positive sensory qualities are the following: savory flavor, spicy flavor, juicy, elastic, tender, smooth texture, chicken meat smell, overall pleasant appearance, characteristic meat aroma, uniform color on the surface, and seasoning in the right amount. The samples that resembled these sensory characteristics were CRBS3 and CRTS5 (spinach-filled samples), as well as CRBC1 and CRTC2 (control samples), which tasters most frequently marked as being present in these samples. In addition to these sensory attributes that have been listed above, characteristic of CRBS3 and CRTS5 samples, there are also uniform filling color, uniform filling distribution, filling adherence to meat, balanced meat/filling taste, and balanced filling flavor intensity, which are at the left middle extreme of the graph, as they only concern the filling of the studied samples, which is not characteristic of the control samples (CRBC1 and CRTC2) but only of the stuffed samples. The samples with chicken offals filling (CRBO4 and CRTO6) are located in the upper left section of the graph and are correlated, in a higher proportion, with the attributes of aftertaste and unevenly distributed filling. They are also correlated, in a slightly lower proportion, with the attributes of metallic taste, low chicken taste, intense filling flavor, excessive seasoning, blood taste, smell of offals filling, and bitter taste. Given that these sensory attributes are located farthest on the graph from the attribute “Overall pleasant product,” which refers to the products’ hedonic score, it can be inferred that consumers valued samples CRBO4 and CRTO6 less than they did the other samples under analysis.

The principal coordinate analysis (PCoA) presented in Figure 2b in graphical form shows the sensory attributes present in the CATA test that were checked by the tasters together in the same sample. By analyzing the PCoA graph, we can observe that the sensory attributes are grouped into five groups. The most favorable attributes checked simultaneously by the tasters in the same sample are those in the upper-right part of the graph, represented by the smell of chicken meat, overall pleasant appearance, savory taste, and pale or undercooked area, which are closest to the favorable hedonic score represented in the graph by the attribute overall pleasant product. The samples CRBC1, CRTC2, CRBS3, and CRTS5 are the ones that, according to Figure 2a, are correlated with these terms. Close to these attributes, positively correlated by the tasters, is the group composed of the sensory terms uniform color on the surface, the smell of spinach filling, tart taste, the taste of spices, elastic, juicy, balanced filling flavor intensity, smooth texture. Among the terms in these first two described groups, we find some of the most favorably positioned terms in the CATA graph (Figure 2a). The next group of sensory attributes that is close to the second group described is the one located at the lower part of the second group and of the graph and presents the sensory attributes that were attributed to CRBS3 and CRTS5 samples in the CATA graph, being represented by the attributes of balanced filling flavor intensity, evenly distributed filling, uniform color of filling, filling adhesion to the meat, and balanced meat/filling taste. The group on the left side of the graph depicts less desirable or undesirable attributes in the analyzed samples, as some of them coincide with attributes that were less appreciated by consumers in the CATA analysis (Figure 2a), e.g., blood taste, dark color (burnt), unevenly distributed filling, and metallic taste, which are attributed according to this analysis to CRBO4 and CRTO6 products, which mainly target taste deficiencies and lead to a generally negative perception of the evaluated products. Located on the left side of the PCoA graph, the final group of sensory attributes includes characteristics like tasteless/low taste intensity, dry, brittle texture, spotty coloration, coarse texture, and under-seasoned. These characteristics primarily relate to the texture of the samples, but they also affect their appearance and taste. They are situated near the control samples (CRBC1 and CRTC2) in the CATA graph (Figure 2a), and on the PCoA graph (Figure 2b), they are on the positive side of the F1 axis but on the negative side of the F2 axis. This indicates that they leave a faintly negative impression on the general perception of these products.

**Figure 3:**
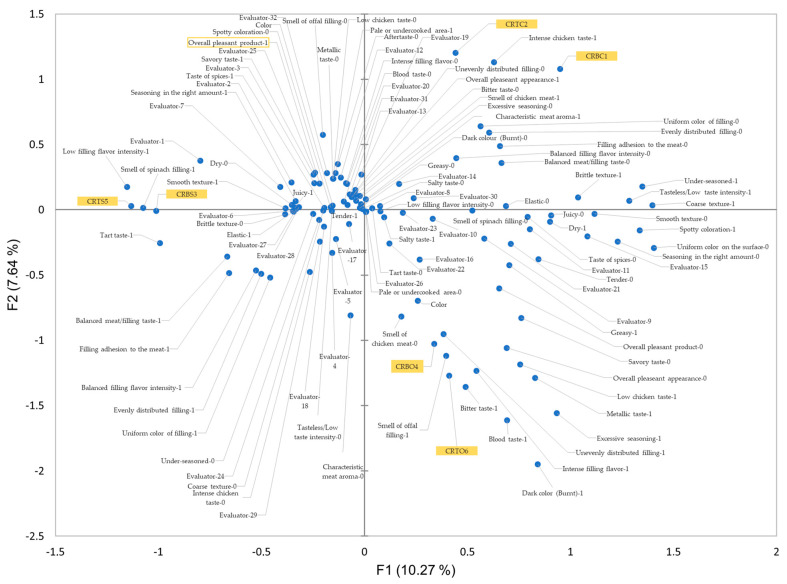
Biplot multiple correspondence analysis (MCA) using the characteristics of CATA analysis, assessors, and product connections in order to establish the correlation between them CRBC1—control chicken roulade made from chicken breast; CRTC2—control chicken roulade made from chicken thigh; CRBS3—chicken roulade made from chicken breast and spinach filling; CRBO4—chicken roulade made from chicken breast and chicken offals filling; CRTS5—chicken roulade made from chicken thigh and spinach filling; CRTO6—chicken roulade made from chicken thigh and chicken offals filling. In addition to the sensory attributes, the values 1 and 0 denote their coding and presence or absence in the analyzed samples. The value ‘1’ denotes the presence of the attribute in the sample or samples near that attribute, while the value ‘0’ denotes the absence of the attribute in the samples near that attribute. Overall pleasant product is the overall score obtained in the hedonic analysis, and its ranking illustrates which sensory most contributed to the greatest hedonic score. Each assessor’s place in the sensory analysis is indicated by the number that appears next to them, which is based on answer order.

The multiple correspondence analysis (MCA) graph, which displays the majority of the CATA analysis’s responses, illustrates the specific features of the filled and control chicken rolls in Figure 3. It also identifies the most specific features for each of the six samples and customers with comparable sensory perceptions. Products with the same type of stuffing or products used as a control are positioned very close to one another, as shown in this figure and Figure 2a. This suggests that their attributes are very similar and are not affected, except in a very small way, by the anatomical region of the chicken carcass from which the rolls were made, but more of filling type or lack of it. As can be seen by analyzing the MCA biplot, the responses for the spinach-stuffed roll samples (CRBS3 and CRTS5) are very balanced across raters, as nearly 40% of the raters ticked the following sensory attributes as being present in these stuffed chicken meat rolls: uniform color on the surface, savory taste, overall pleasant product, juicy, elastic, smooth texture, low filling flavor intensity, smell of spinach filling, filling adhesion to the meat, tart taste, balanced meat/filling taste, taste of spices, tender, and overall pleasant appearance. The presence of the number 1 next to the sensory qualities indicates that they are present in the samples. Tasteless/low taste intensity, dry, coarse texture, salty taste, under-seasoned, intense chicken taste, spotty coloration, blood taste, bitter taste, aftertaste, intense filling taste, and metallic taste were among the characteristics that were not checked as not being specific to CRBS3 and CRTS5, which were marked as absent (sensory attributes are accompanied by the digit 0). Since these sensory properties were positioned at roughly identical distances on the graph between these two groups of chicken roulades, most of these characteristics—both those indicated as present and those marked as absent—were likewise present in the control chicken roulades (CRBC1 and CRTC2). From this we can deduce the significant similarities between these four chicken roulades of evidence in terms of qualitative attributes. The sensory characteristics of offals-filled chicken roulades (CRBO4 and CRTO6) vary significantly from those of the other roll types examined in this study. This imbalance in sensory quality may account for the lower hedonic scores these products received. Most consumers have identified characteristics like metallic taste, blood taste, unevenly distributed filling, excessive seasoning, low chicken taste, intense filling flavor, dark color (burnt), aftertaste, salty taste, and dryness in these products (Table 4). These notes are also caused by the absence of specific sensory qualities that customers want, such as the distinct scent of meat, the smell of chicken meat, the savory taste, the tenderness, and the taste of spices. The main cause of customer discontent is the flavor of the offals, which is a strong overlay of the distinctive meat aroma. It is evident that many of these features are connected to the aroma of chicken meat.

#### 3.1.3. Quantitative Descriptive Analysis

The primary sensory characteristics of the items were not covered in the hedonic analysis; thus, we chose to address them in the descriptive quantitative analysis for every sensory attribute found in the CATA test. With the exception of the sensory attributes of spotty coloration and excessive seasoning, which vary insignificantly (*p* > 0.05) between the samples examined, practically all of the 40 sensory attributes scored with the quantitative data collected from the 32 raters (Table 5) vary highly significantly (*p* < 0.001). The mean values in Table 5 show that the differences between the control samples (CRBC1 and CRTC2) and the spinach-filled chicken roulade samples (CRBS3 and CRTS5) are not statistically significant. However, there is a significant difference between these four samples and the chicken roulade samples that were filled with chicken offals (CRBO4 and CRTO6).

**Figure 4:**
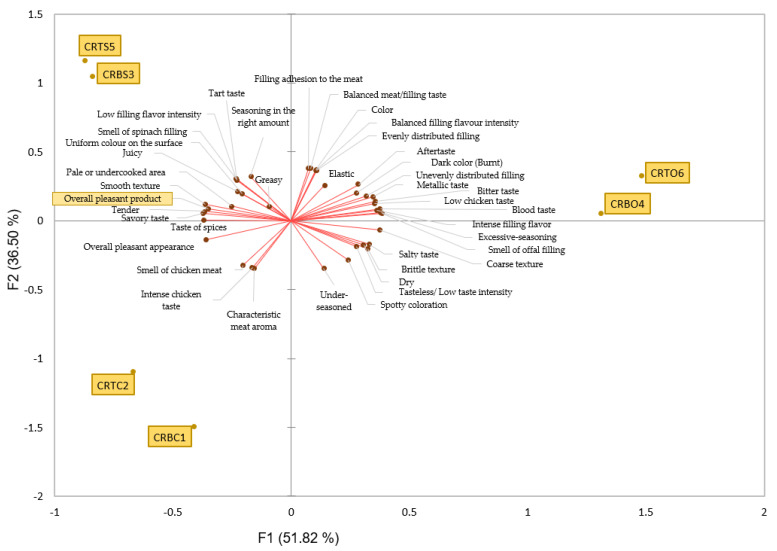
Relationships between sensory attributes represented using principal component analysis (PCA). CRBC1—control chicken roulade made from chicken breast; CRTC2—control chicken roulade made from chicken thigh; CRBS3—chicken roulade made from chicken breast and spinach filling; CRBO4—chicken roulade made from chicken breast and chicken offals filling; CRTS5—chicken roulade made from chicken thigh and spinach filling; CRTO6—chicken roulade made from chicken thigh and chicken offals filling. The samples associations with adjacent sensory attributes—shown by red arrows—determine where they appear on the PCA plot. Overall pleasant product is the overall score obtained in the hedonic analysis, and its ranking illustrates which sensory most contributed to the greatest hedonic score. Each assessor’s place in the sensory analysis is indicated by the number that appears next to them, which is based on answer order.

Principal component analysis (PCA) is one method that can provide a broad overview of the complexities and connections found in multivariate data sets. The quantitative results in the table for the sensory qualities are visually distributed and correlated in the PCA plot, which is shown in Figure 4. On the graph’s right side, the sensory characteristics that are located in the top quadrant are represented by coarse texture, smell of offals filling, excessive seasoning, intense filling flavor, blood taste, low chicken taste, bitter taste, metallic taste, unevenly distributed filling, dark color (burnt), aftertaste, elastic, evenly distributed filling, balanced filling flavor intensity, uniform color of filling, balanced meat/filling taste, and filling adhesion to the meat. The first half described above is graphically specific to CRBO4 and CRTO6. Even though CRBO4 and CRTO6 are in the upper right quadrant, which is correlated with both the F1 and F2 axes, this does not necessarily mean that consumers prefer the attributes found there. Despite being widely used and documented by many authors in a variety of applications, PCA cannot be considered a categorization approach because it is typically used to extract and compress multivariate data sets, identify and quantify patterns and trends, discover outliers, and reveal relationships between variables and samples (e.g., patterns) [41]. This leads us to the conclusion that the PCA analysis only shows the positive, negative, or neutral correlations between the sensory qualities rather than the values of the attributes themselves. Therefore, we can see that several of the characteristics in the top right quadrant—from coarse texture to aftertaste—were specific to CRBO4 and CRTO6 products as well as to the investigations carried out thus far in this research. Additionally, the favorable sensory attributes in the upper right quadrant, which are near the F2 axis, form an angle of about 180 degrees with the overall pleasant product sensory attribute, demonstrating a negative correlation with them. This indicates that the scores assigned to the sensory attributes in the upper left quadrant vary inversely proportionally with those in the upper right quadrant.

Together with the characteristics of the CRBS3 and CRTS5 samples—taste of spices, savory taste, tenderness, smooth texture, pale or undercooked area, uniform color on the surface, juiciness, and smell of spinach filling—the attribute of an overall pleasant product, which refers to the favorable hedonic score of the analyzed products, is situated in the upper left quadrant of the PCA graph (Figure 4). As they form an angle < 60°, they are perceived as moderately positive. In addition to these highly favorable product attributes of CRBS3 and CRTS5, the same quadrant also contains the following: tart taste, low filling flavor intensity, greasy, and seasoning in the right amount. These also exhibit a very slight negative correlation with the sensory attributes in the lower area of this quadrant. The lower left quadrant of the PCA plot contains the control samples CRBC1 and CRTC2, which are distinguished by their characteristic meat aroma, intense chicken taste, and overall pleasant appearance. These characteristics are highly specific to these samples because the filling is not present, and the sensory aspects of chicken meat predominate the analysis.

**Figure 5:**
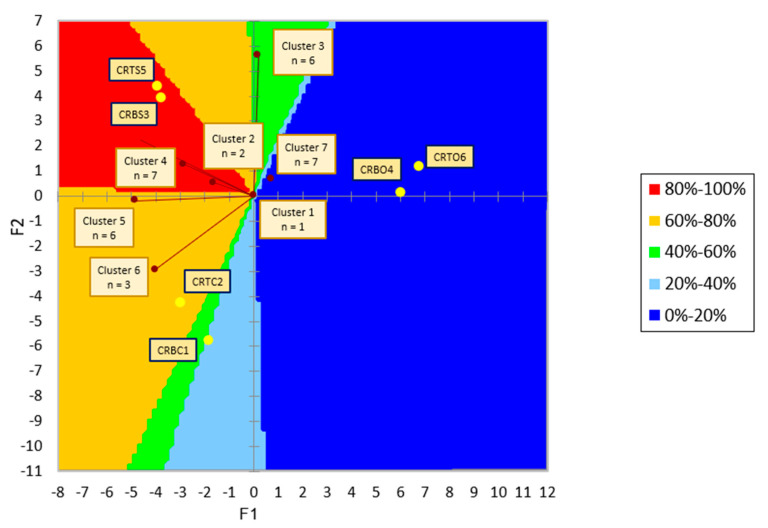
PrefMap graph for sensory profiles and assessor preferences for chicken roulades. CRBC1—control chicken roulade made from chicken breast; CRTC2—control chicken roulade made from chicken thigh; CRBS3—chicken roulade made from chicken breast and spinach filling; CRBO4—chicken roulade made from chicken breast and chicken offals filling; CRTS5—chicken roulade made from chicken thigh and spinach filling; CRTO6—chicken roulade made from chicken thigh and chicken offals as filling. The legend on the right side of this graphic illustrates how the colors on the backdrop represent the preference classes as percentage values. Based on their responses to the sensory evaluation, the consumers are grouped into clusters, and the letter n indicates how many assessors are in each cluster.

The PrefMap graph, presented in Figure 5, is a representation of consumer preferences regarding the analyzed chicken roulades. Customers’ preferences can be linked to certain sensory aspects of the products using the external preference mapping approach. This strategy is important because it provides a solid foundation for modifying or producing goods that meet customer expectations. The initial interpretation of the graph refers to the positioning of the six samples analyzed (CRBC1, CRTC2, CRBS3, CRBO4, CRTS5, and CRTO6) in different areas of the graph with different colorations, which refer to the degree of consumer preference and acceptability of the stuffed chicken roulades, which are new products for consumers, and the further interpretation is made by analyzing the position of the clusters in relation to the products analyzed, which highlight the degree of similarity or differentiation between consumer preferences. The colors of the graph represent a map of the evaluators’ preferences, which signify the percentage of consumer preference: 0% represents the lowest level of preference, and 100% represents the highest level of preference. According to these meanings, we can observe that the products located in the red color area of the graph are the most appreciated, being preferred by more than 80% of the evaluators, which are represented by chicken roulades filled with spinach (CRBS3 and CRTS5). In terms of appearance, texture, olfactory perception, taste, and overall liking of the samples, their position indicates that they most fully satisfy the criteria of consumers’ sensory preferences for the evaluators in Clusters 4 and 2, but we can also see a tendency toward these samples from the evaluators in Cluster 4 and even Cluster 3 to a lesser degree. The chicken roulades filled with chicken offals (CRBO4 and CRTO6) sit in the blue section of the graph, indicating that they scored the lowest on the sensory attributes and, consequently, had a lower hedonic score than all the other samples examined. This contrasts with the chicken roulades filled with spinach (CRBS3 and CRTS5). The fact that the CRBO4 and CRTO6 samples are not close to the raters’ clusters indicates that the raters did not especially like them, although some consumers, such as those in Clusters 1 and 7, likewise showed little interest in them. Additionally, they are a considerable distance from the other samples that the panelists assessed and scored, suggesting that these products have sensory qualities that differ greatly from those of the other chicken roulade samples that were examined.

The control samples CRBC1 and CRTC2 present in the PrefMap graph (Figure 5) are located in different color areas of the graph, which means that they are perceived differently depending on the anatomical region from which the chicken roulade is made. The CRTC2 sample, which represents the control chicken roulade made without a filling, is preferred by 60–80% of the consumers, indicating that the specific attributes of chicken meat are more preferred for this type of product, as observed in the liking (hedonic) test. Consumers in Cluster 5 and Cluster 6 liked this product the most and rated it as preferable to the others. The control chicken roulade made from the chicken breast (CRBC1) showed a decreased consumer preference compared to the other control roulade (CRTC2), being positioned in an intermediate position between two different color areas (green and light blue) of the PrefMap graph and being preferred by about one-third of the consumers. This sample is not specifically preferred by any group of consumers, not polarizing any cluster in the CRBC1 sample area, but nevertheless, it is quite close to cluster 6, signaling some preference of the evaluators in this cluster for this sample.

**Figure 6:**
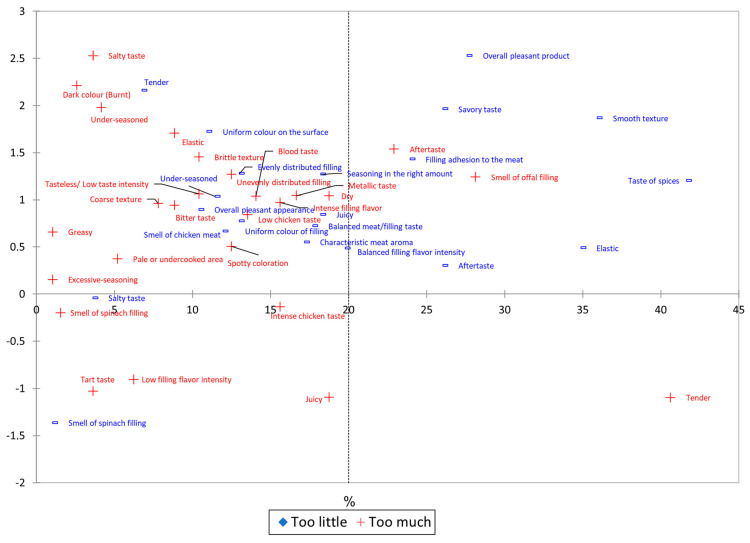
Penalty analysis mean drop plots for all six samples of chicken roulades. The graph displays the sensory attributes that consumers believe to be “too little” (blue) or “too much” (red) in relation to what they believe to be the ideal quantity of those attributes. Depending on its degree and the panelists’ personal preferences, the same trait may be viewed as either an excess (too much) or a deficit (too little).

In order to better understand consumer preferences for the chicken roulades under investigation, we used penalty chacteristics that influenced the sensory preference score of the samples under study from the viewpoint of the consumers (Table 6 and Figure 6). Only the sensory features that exhibit consumer penalties are examined in Table 6, not the full set of 40 sensory attributes that the panel identified and examined. Figure 6 illustrates the impact of additional sensory qualities on the overall score of the examined samples.

Penalty analysis highlights the sensory attributes that contribute to the decrease in overall consumer satisfaction (Table 6). The highest penalties are recorded for the sensory attributes of salty taste (1.242), under-seasoned (1.285), and aftertaste (0.880). These are the characteristics that decrease consumer preferences the most and lead to a low hedonic score. In addition, the sensory attributes of tenderness (−0.632), elasticity (0.734), juiciness (−0.140), and smell of spinach filling (−0.666) also lead to a penalization of the studied chicken rolls. The highly significant decreases (*p* < 0.001) on the hedonic score are recorded for salty taste, tenderness, elasticity, aftertaste, and under seasoning, while for the attributes juicy and smell of spinach filling the influence on the overall enjoyment of the products is insignificant (*p* > 0.05), as the scores for these characteristics deviate very little from the JAR level. These last two characteristics, as observed in the sensory evaluations carried out so far, are specific to spinach-filled chicken roulades (CRBS3 and CRTS5); hence, it can be deduced why they enjoyed scores among panelists indicating high sensory liking of these products on the part of panelists and a general score in terms of overall liking of the product, as they show insignificant deviations from the JAR. Attributes showing significant penalizations leading to highly significant (*p* < 0.001) penalties on the final score of chicken meat roulades, as can be observed in this research, are specific to roulades with animal fillings (chicken offals), coded CRBO4 (chicken breast roulade with offals) and CRTO6 (chicken breast roulade and chicken thigh with offals), these results explaining the low popularity among panelists of these products.

The sensory characteristics shown in Figure 6 demonstrate how customers’ perceptions of chicken roulades are impacted when the sensory characteristics are not ideal (JAR). This chart illustrates the impact of sensory attribute deviations from JAR and their impact on the hedonic score rather than the fines imposed on the product. Since even if the scores obtained within the QDA deviated from JAR, these deviations were either very close to JAR or the average of consumers who reported these deviations was very low, the absence of these sensory characteristics in Table 6 suggests that they do not significantly penalize the analyzed samples. Sensory attributes at the extremes of the graph, e.g., salty taste, overall pleasant product, smooth texture, taste of spices, elastic, tender, juicy, smell of spinach filling, low filling flavor intensity, tart taste, excessive seasoning, greasy, under-seasoned, dark color (burnt) showed quite frequent deviations from the JAR, being recorded by a large number of panelists, but, nevertheless, not all of these characteristics scores negatively affected the panelists’ preferences. The smooth texture, taste of spices, savory taste, and smell of spinach filling are examples of attributes that panelists frequently reported as “not enough” (these are shown on the graphic by the presence of a minus sign and a blue color). These attributes did not result in penalties in the overall appreciation of the products because, even though the deviations were numerous, they were not far from the JAR. The same is true for characteristics that are listed as “too much”, such as dark color (burnt), greasy, excessive seasoning, smell of spinach filling, tart taste, and low filling flavor intensity. However, there are also characteristics at the extremes of the graph, such as salty taste, tenderness, juiciness, elasticity, under seasoning, and smell of spinach filling, perceived by the panelists as “not enough”, “too much”, or both, which present deviations from the hedonic score, sometimes leading to extremely significant decreases (*p* < 0.001) of chicken roulades, or insignificant (*p* > 0.05), as is the case for the sensory attributes juicy and smell of spinach filling, as can be seen in Table 6.

Sensory attributes positioned in the middle area of Figure 6, e.g., intense chicken taste, spotty coloration, balanced filling flavor intensity, low chicken taste, blood taste, brittle texture, unevenly distributed filling, seasoning in the right amount, uniform color of filling, bitter taste, coarse texture, intense filling flavor, metallic taste, tasteless/low taste intensity, characteristic meat aroma, smell of chicken meat, and uniform color on the surface, show fewer deviations from JAR; yet, because there are no penalties, even though some of them may be many, they do not significantly affect the chicken roulades’ hedonic score. The sensory attributes positioned in the middle area of Figure 6 generally reflect an acceptable organoleptic profile, with no significant deviations influencing the overall consumer perception. For example, characteristics such as moderate chicken taste, slightly non-uniform coloring, or balanced intensity of the filling flavor suggest a level of sensory homogeneity. However, the presence of negative attributes, such as bitter taste, coarse texture, or chicken-like odor, may indicate subtle variations in the quality of the ingredients or the technological process. Since no penalties are applied for these deviations, their impact on the overall hedonic score of the chicken roulade remains limited. This observation emphasizes that, in the context of sensory evaluation, consumers tend to place greater importance on dominant attributes at the expense of less intense or less frequent ones. Thus, the chicken roll manages to maintain overall acceptability, even if some characteristics could benefit from improvements to optimize sensory quality.

## 4. Discussion

Very little information about meat products, like chicken roulades with fillings, can be found in the specialized literature, and our search turned up no articles discussing this subject in general or sensory analysis in particular, and this is the first study to examine chicken roulades filled with spinach and chicken offals. However, studies on the addition of spinach in meat products, especially chicken meat, have been carried out by Carvalho et al. [42] and Kim [43] on chicken burgers and beef hamburgers, respectively, by Castenmiller et al. [44] on meatballs, by Vasyleva et al. [45] on poultry semi-finished products, and by Aly and Morsy [46] on chicken burgers s.a., but these did not focus on the sensory analysis of these products in such a detailed way or not at all, emphasizing only the nutritional value of the products. The trend to add vegetable ingredients to chicken meat can be observed, as consumers tend to see this type of meat as having a beneficial nutritional composition due to its low saturated fat content, and it can also be very easily fortified with nutrients from a variety of sources [47].

Using sensory analysis and, especially, the PrefMap analysis (Figure 5), it was found that the spinach-filled roulade batches (CRBS3 and CRTS5), regardless of the type of meat used, enjoyed the highest appreciation from the panelists, even surpassing the control batches (CRBC1 and CRTC2) with which consumers are generally most familiar. However, the chicken roulade batches that were filled with offals (CRBO4 and CRTO6) were very poorly appreciated by consumers, a fact also observed by Llauger et al. [48], where a large number of study participants expressed disapproval of meat products that contained offals extracts, mostly due to health and sensory issues. Sensorial characteristics are a driver of food preferences, which explains why many consumers are unwilling to forgo the enjoyable experience that a product offers, even if it has additional benefits. Analyzing this information, we can deduce that spinach-filled chicken roulades (CRBS3 and CRTS5), regardless of the anatomical region used for their manufacture, enjoy high success among consumers, being even superior to the classic chicken roulade formulations, without filling, with which consumers are already familiar due to the presence of this type of product on the market. This fact shows a high consumer acceptability, in general, for meat products with vegetable additives, even paving the way for new hybrid meat-vegetable products.

The reformulation of some meat products with offals is not very popular, as seen by the scarcity of research studies on the subject. However, we have identified articles in which edible offals are fraudulently added to replace meat, as seen in the work of Banu and Șmatoc [49] or in the study by Llauger et al. [48], which analyzed consumer perceptions of reforming some meat products with offals in order to provide a positive response to the increasing presence of animal protein requirements in the human diet. According to these sources, offals are not very popular among consumers, as evidenced by our study (CRBO4 and CRTO6), which consistently scored the lowest in all sensory tests. Even while there are products derived from offals that are traditional and highly valued in certain nations, as we can see from the study of Babicz et al. [50], they are not as appreciated in geographical locations where there is no tradition of their consumption. This could be related to people’s predisposition to avoid unfamiliar food products for a variety of reasons, including a fear of eating poisons; this tendency is known as neophobia, and it is stronger for animal products than non-animal products [51].

## 5. Conclusions

The use of sensory analysis in meat science is growing in popularity as a vital technique for creating novel meat products. A thorough explanation of how a food item is experienced by the human senses and/or how much it is loved are two metrics that sensory science, when used properly, offers that no other instrument (to date) can measure. Therefore, the development of current meat products or the diversification of their variety range requires careful consideration of the width of any sensory testing and acceptable sensory practices.

With its unique sensory profile, together with other qualities, meat and meat products play a significant role in human nutrition. The consumption of meat and meat products rises in tandem with society’s level of development and standard of life. The goal of the current study was to create new meat products that would meet the ever-changing demands of the modern consumer, who wants greater or healthier products without sacrificing their sensory qualities—possibly even better ones that specifically target meat products—and adjust to the volatile food market of recent years. Additionally, we would like to introduce a novel way to use some foods, like spinach, but particularly offals, as consumers, particularly young consumers, do not value offals despite their nutritious benefits. In order to produce a valuable product that is better than what is already available, we set out to fortify low-iron meat with both animal and plant sources of iron. We also wanted to examine consumer perception in order to gauge the product’s sensory quality. In order to determine whether consumer tastes for chicken roulades varied depending on the anatomical region used, rather than merely the filling, we made four batches, using chicken breast meat in two and chicken thigh in the other two. Separately, these roulades were filled with either spinach or chicken offals (liver, heart, and gizzard). In order to be comparable with a well-known, recognizable product, two chicken roulades were also prepared as control samples, devoid of filling. The hedonic test revealed that the chicken breast roll with spinach filling (CRBS3) was less popular than its chicken thigh counterpart, indicating that chicken breast had a minor but significant impact on consumer preferences for chicken rolls. The control chicken roulade samples (CRBC1 and CRTC2), on the other hand, demonstrated the same degree of consumer appreciation on the hedonic scale (over 7 points), regardless of the anatomical region used to make them. There was a larger, significant difference in the anatomical region used for the chicken roulade; however, according to the PrefMap analysis, which also examined the individual scores of all sensory qualities and the hedonic test scores in greater detail. Even between the control sample chicken roulade made from chicken thigh (CRTC2) and the same sample made from chicken breast (CRBC1), there was a fairly significant difference, with 40–60% of consumers preferring the former and only roughly half preferring the latter. The batches of chicken roulades filled with chicken offals (liver, hearts and gizzards), labeled CRBO4 and CRTO6, were the only samples in which samples created from chicken breasts were valued more highly than samples made from chicken thighs. This tendency is unique to our study; in this instance, the batch of chicken rolls prepared from chicken thighs (CRTO6) received lower ratings than the batch made from chicken breasts (CRBO4).

Based on our research, we can say that spinach-filled chicken roulades are an appealing option for the future of iron-enriched hybrid meat products. They are positively welcomed by customers, even outperforming traditional chicken roulades, which opens the door for the meat business to innovate and become more competitive. Offals has inherent virtues, but unfortunately, it is not the best choice for innovation in the meat sector. The less appealing sensory qualities that they impose on food products—such as their distinct flavor, texture, and scent—are indelible to consumers and, as we have seen, do not fit into their preferences.

## Figures and Tables

**Table 1:** The components of the roulades and the percentages of each in the samples under study.

Sample	Chicken Breast, %	Chicken Thigh, %	Filling, %
CRBC1	100	-	-
CRTC2	-	100	-
CRBS3	70	-	30
CRBO4			
CRTS5	-	70	
CRTO6			

CRBC1—control chicken roulade made from chicken breast; CRTC2—control chicken roulade made from chicken thigh; CRBS3—chicken roulade made from chicken breast and spinach filling; CRBO4—chicken roulade made from chicken breast and chicken offals filling; CRTS5—chicken roulade made from chicken thigh and spinach filling; CRTO6—chicken roulade made from chicken thigh and chicken offals filling. The same spice blend, which includes salt, black pepper, pink pepper, green pepper, sweet paprika, and garlic powder, was used in every batch of roulades.

**Table 2:** The chicken roulades’ thermal treatment parameters.

Stage	Time (min.)	Temperature (°C)	The Temperature in the Center of the Product (°C)	Humidity (%)
Drying	30–50	65	55	25
Smoking	30–60	65	55	25
Boiling	120	72	69	99
Drying	20	80	72	25
Roasting	10–20	80–95	72	22

**Table 3:** CATA test sensory qualities evaluated by tasters.

Sensory Property	CATA Terms
Appearance	overall pleasant appearance, pale or undercooked area, dark color (burnt), uniform color on the surface, uniform color of filling, spotty coloration, evenly distributed filling, unevenly distributed filling;
Texture	dry, juicy, smooth texture, brittle texture, coarse texture, elastic, tender, greasy, filling adhesion to the meat;
Olfactive perception	the smell of chicken meat, the smell of spinach filling, the smell of offals filling;
Taste	characteristic meat aroma, low filling flavor intensity, balanced filling flavor intensity, intense filling flavor intensity, salty taste, under-seasoned, seasoning in the right amount, excessive seasoning, metallic taste, blood taste, tart taste, bitter taste, tasteless/low taste intensity, low chicken taste, balanced meat/filling taste, intense chicken taste, savory taste, a taste of spices, aftertaste;
Overall liking	overall pleasant product.

**Table 4:** Sensory characteristics that were examined and the frequency with which they were verified to be present in each of the examined samples.

Sensory Attributes	Frequency of Presence	Cochran’s Q Test *p*-Value and Significance	Sensory Attributes	Frequency of Presence	Cochran’s Q Test *p*-Value and Significance
Overall pleasant appearance	176	**0.003**	Metallic taste	30	**<0.0001**
Pale or undercooked area	10	0.416	Spotty coloration	21	**0.024**
Dark color (Burnt)	4	**0.001**	Uniform color on the surface	173	**0.035**
Uniform color of filling	106	**<0.0001**	Elastic	128	**<0.0001**
Evenly distributed filling	105	**<0.0001**	Tender	175	**0.016**
Unevenly distributed filling	22	**<0.0001**	Greasy	6	**0.053**
Characteristic meat aroma	175	**0.013**	Filling adhesion to the meat	96	**<0.0001**
Balanced filling flavor intensity	88	**<0.0001**	Low chicken taste	32	**<0.0001**
Low filling flavor intensity	12	**<0.0001**	Balanced meat/filling taste	96	**<0.0001**
Intense filling flavor	27	**<0.0001**	Intense chicken taste	57	**<0.0001**
Salty taste	19	0.691	Tart taste	7	**0.001**
Tasteless/low taste intensity	21	**0.000**	Blood taste	21	**<0.0001**
Under-seasoned	30	**<0.0001**	Bitter taste	15	**<0.0001**
Seasoning in the right amount	151	**0.000**	Savory taste	145	**<0.0001**
Excessive seasoning	8	**0.007**	Smell of chicken meat	170	**0.015**
Dry	54	**<0.0001**	Smell of spinach filling	63	**<0.0001**
Juicy	140	**<0.0001**	Smell of offals filling	65	**<0.0001**
Smooth texture	143	**<0.0001**	Taste of spices	154	**<0.0001**
Brittle texture	52	**0.001**	Overall pleasant product	144	**<0.0001**
Coarse texture	24	**0.003**	Aftertaste	64	**<0.0001**

Attributes that differentiate the samples significantly are highlighted in bold.

**Table 5:** Means and standard deviations for the sensory characteristics of the examined chicken roulades samples.

Sensory Attributes	CRBC1	CRTC2	CRBS3	CRBO4	CRTS5	CRTO6	*p*-Value
Salty taste	4.84 ± 0.13	5.03 ± 0.15	4.28 ± 0.13	5.53 ± 0.16	4.34 ± 0.12	5.28 ± 0.18	<0.0001
Tender	7.22 ± 0.19	6.97 ± 0.21	7.44 ± 0.22	5.91 ± 0.30	8.00 ± 0.15	5.69 ± 0.29	<0.0001
Elastic	3.41 ± 0.21	4.34 ± 0.18	4.28 ± 0.22	4.84 ± 0.17	6.25 ± 0.21	6.28 ± 0.17	<0.0001
Coarse texture	1.72 ± 0.18	1.38 ± 0.16	1.28 ± 0.14	2.34 ± 0.22	1.29 ± 0.07	2.22 ± 0.20	<0.0001
Aftertaste	2.00 ± 0.23	2.00 ± 0.21	3.97 ± 0.15	5.78 ± 0.18	4.41 ± 0.12	5.84 ± 0.14	<0.0001
Juicy	4.41 ± 0.18	6.19 ± 0.25	6.88 ± 0.20	3.28 ± 0.30	7.53 ± 0.13	6.03 ± 0.33	<0.0001
Dry	2.78 ± 0.15	2.13 ± 0.22	1.59 ± 0.19	3.78 ± 0.34	1.16 ± 0.08	2.72 ± 0.29	<0.0001
Greasy	1.00 ± 0.00	1.56 ± 0.11	1.00 ± 0.00	1.00 ± 0.00	1.88 ± 0.14	1.44 ± 0.14	<0.0001
Brittle texture	2.34 ± 0.23	1.81 ± 0.20	1.25 ± 0.08	2.56 ± 0.18	1.13 ± 0.06	2.59 ± 0.18	<0.0001
Uniform color on the surface	7.53 ± 0.22	7.75 ± 0.22	8.69 ± 0.10	8.06 ± 0.17	8.13 ± 0.15	7.06 ± 0.22	<0.0001
Spotty coloration	2.56 ± 0.25	2.47 ± 0.17	2.00 ± 0.13	2.47 ± 0.22	2.13 ± 0.17	2.59 ± 0.23	0.089
Pale or undercooked area	2.28 ± 0.17	1.06 ± 0.06	2.50 ± 0.18	1.38 ± 0.12	2.19 ± 0.16	1.00 ± 0.00	<0.0001
Dark color (Burnt)	1.09 ± 0.09	1.00 ± 0.00	1.34 ± 0.09	1.53 ± 0.12	1.56 ± 0.12	2.31 ± 0.23	<0.0001
Uniform color of filling	-	-	7.91 ± 0.20	7.22 ± 0.20	7.84 ± 0.16	6.78 ± 0.21	<0.0001
Overall pleasant appearance	8.66 ± 0.12	8.66 ± 0.12	8.41 ± 0.13	7.16 ± 0.18	8.19 ± 0.16	7.09 ± 0.20	<0.0001
Evenly distributed filling	-	-	7.88 ± 0.26	7.53 ± 0.20	7.81 ± 0.24	6.56 ± 0.23	<0.0001
Unevenly distributed filling	-	-	1.63 ± 0.24	2.09 ± 0.26	1.53 ± 0.21	3.16 ± 0.26	<0.0001
Characteristic meat aroma	9.00 ± 0.00	9.00 ± 0.00	6.90 ± 0.23	6.88 ± 0.18	6.98 ± 0.18	6.88 ± 0.15	<0.0001
Low chicken taste	1.00 ± 0.00	1.00 ± 0.00	1.63 ± 0.21	2.75 ± 0.26	1.13 ± 0.07	2.53 ± 0.28	<0.0001
Balanced meat/filling taste	-	-	8.06 ± 0.20	6.78 ± 0.21	8.06 ± 0.17	6.69 ± 0.22	<0.0001
Intense chicken taste	7.59 ± 0.15	8.81 ± 0.10	1.19 ± 0.08	1.00 ± 0.00	1.50 ± 0.16	1.00 ± 0.00	<0.0001
Savory taste	7.31 ± 0.21	7.88 ± 0.14	7.84 ± 0.19	6.72 ± 0.26	8.09 ± 0.18	6.34 ± 0.23	<0.0001
Tasteless/Low taste intensity	2.34 ± 0.25	1.19 ± 0.10	1.06 ± 0.04	2.34 ± 0.30	1.03 ± 0.03	2.00 ± 0.22	<0.0001
Tart taste	1.00 ± 0.00	1.00 ± 0.00	1.88 ± 0.23	1.00 ± 0.00	1.63 ± 0.16	1.00 ± 0.00	<0.0001
Blood taste	1.00 ± 0.00	1.00 ± 0.00	1.13 ± 0.07	2.34 ± 0.28	1.13 ± 0.06	2.66 ± 0.28	<0.0001
Smooth texture	6.90 ± 0.21	7.63 ± 0.25	8.09 ± 0.21	5.88 ± 0.30	8.31 ± 0.16	6.03 ± 0.23	<0.0001
Under-seasoned	3.13 ± 0.34	2.31 ± 0.36	1.38 ± 0.12	2.50 ± 0.23	1.31 ± 0.10	2.16 ± 0.28	0.000
Seasoning in the right amount	6.38 ± 0.34	7.34 ± 0.38	8.06 ± 0.10	6.94 ± 0.19	8.06 ± 0.10	7.19 ± 0.26	<0.0001
Excessive seasoning	1.00 ± 0.00	1.00 ± 0.00	1.00 ± 0.00	1.09 ± 0.09	1.00 ± 0.00	1.13 ± 0.13	0.475
Low filling flavor intensity	-	-	1.75 ± 0.14	1.00 ± 0.00	1.88 ± 0.24	1.00 ± 0.00	<0.0001
Balanced filling flavor intensity	-	-	7.78 ± 0.19	6.72 ± 0.26	7.53 ± 0.25	6.97 ± 0.22	<0.0001
Intense filling flavor	-	-	1.09 ± 0.09	2.88 ± 0.32	1.19 ± 0.14	2.25 ± 0.22	<0.0001
Bitter taste	1.00 ± 0.00	1.00 ± 0.00	1.25 ± 0.10	1.72 ± 0.18	1.16 ± 0.07	1.94 ± 0.21	<0.0001
Metallic taste	1.00 ± 0.00	1.00 ± 0.00	1.50 ± 0.12	2.56 ± 0.26	1.56 ± 0.12	2.69 ± 0.28	<0.0001
Smell of chicken meat	9.00 ± 0.00	9.00 ± 0.00	7.69 ± 0.18	7.31 ± 0.16	7.44 ± 0.16	7.19 ± 0.16	<0.0001
Smell of spinach filling	-	-	6.31 ± 0.10	1.00 ± 0.00	6.69 ± 0.11	1.00 ± 0.00	<0.0001
Smell of offals filling	-	-	1.00 ± 0.00	7.16 ± 0.14	1.00 ± 0.00	7.13 ± 0.12	<0.0001
Filling adhesion to the meat	-	-	7.78 ± 0.15	6.00 ± 0.20	7.22 ± 0.12	6.22 ± 0.22	<0.0001
Overall pleasant product	7.22 ± 0.15	7.29 ± 0.18	7.75 ± 0.19	6.78 ± 0.28	7.94 ± 0.21	5.94 ± 0.27	<0.0001
Taste of spices	4.59 ± 0.18	4.03 ± 0.19	4.75 ± 0.17	2.78 ± 0.12	4.53 ± 0.14	2.75 ± 0.12	<0.0001

CRBC1—control chicken roulade made from chicken breast; CRTC2—control chicken roulade made from chicken thigh; CRBS3—chicken roulade made from chicken breast and spinach filling; CRBO4—chicken roulade made from chicken breast and chicken offals filling; CRTS5—chicken roulade made from chicken thigh and spinach filling; CRTO6—chicken roulade made from chicken thigh and chicken offals filling. The ANOVA test and the Tukey post hoc test were used to examine group differences. Significant differences between groups are indicated by *p* < 0.05.

**Table 6:** Penalty analysis table for the chicken roulades samples.

Variable	Level	Frequencies	%	Mean Drops	Penalties	*p*-Value
	Not enough	7	3.65%	−0.044		
Salty taste	JAR	178	92.71%		1.242	**0.001**
	Too much	7	3.65%	2.527		
	Not enough	13	6.77%	2.159		
Tender	JAR	101	52.60%		−0.632	**0.001**
	Too much	78	40.63%	−1.097		
	Not enough	67	34.90%	0.487		
Elastic	JAR	108	56.25%		0.734	**0.000**
	Too much	17	8.85%	1.708		
	Not enough	50	26.04%	0.302		
Aftertaste	JAR	98	51.04%		0.880	**<0.0001**
	Too much	44	22.92%	1.536		
	Not enough	35	18.23%	0.842		
Juicy	JAR	121	63.02%		−0.140	0.501
	Too much	36	18.75%	−1.095		
	Not enough	22	11.46%	1.034		
Under-seasoned	JAR	162	84.38%		1.285	**<0.0001**
	Too much	8	4.17%	1.977		
	Not enough	2	1.04%	−1.366		
Smell of spinach filling	JAR	187	97.40%		−0.666	0.291
	Too much	3	1.56%	−0.200		

ANOVA and Tukey post hoc tests were used to examine group differences. Significant differences between groups are indicated by *p* < 0.05 and are highlighted in bold.

## Data Availability

The original contributions presented in the study are included in the article, further inquiries can be directed to the corresponding author.
